# New pricing models for generic medicines to ensure long-term sustainable competition in Europe

**DOI:** 10.3389/fphar.2023.1200641

**Published:** 2023-10-09

**Authors:** Clement Francois, Gabriela Gawlik, Jorge Mestre-Ferrandiz, Adrian Pana, Julian Perelman, John Yfantopoulos, Steven Simoens

**Affiliations:** ^1^ Aix Marseille University, Marseille, France; ^2^ Putnam PHMR, Paris, France; ^3^ Putnam PHMR, Krakow, Poland; ^4^ Universidad Carlos III, Madrid, Spain; ^5^ Department of Public Health, Babes Bolyai University, Cluj Napoca, Romania; ^6^ NOVA National School of Public Health, Public Health Research Centre, Comprehensive Health Research Center, CHRC, NOVA University Lisbon, Lisbon, Portugal; ^7^ IPOKE Research Institute, MBA-University of Athens, Athens, Greece; ^8^ KU Leuven Department of Pharmaceutical and Pharmacological Sciences, Leuven, Belgium

**Keywords:** generic medicines, pricing, competition, sustainability, Europe

## Abstract

**Background:** Price erosion of generic medicines over time as a result of existing pricing policies in combination with increasing operational costs of these products due to high inflation, undermine long-term sustainable competition in European off-patent medicines markets. Therefore, the aim of this study is to identify new potential pricing models for retail generic medicines in Europe, examine their pros and cons, and illustrate them with examples inside or outside the pharmaceutical sector.

**Methods:** A targeted literature review, one-to-one interviews and a joint advisory board meeting with experts from five European countries were carried out to assess potential pricing models for generic medicines.

**Results:** We identified ten pricing models that can be applied to generic medicines. The tiered pricing model is viewed as a sustainable solution ensuring competitiveness, but requires market monitoring using a supportive IT infrastructure. De-linking the price of generic medicines from that of the off-patent originator medicine prevents the originator from forcing generic medicines’ prices to unsustainable levels. Higher costs due to inflation can be compensated in the automatic indexation model. Other pricing models that have less implementation potential include the one-in-one/multiple-out model, tax credits, value-based pricing, volume for savings and guaranteed margin/fee models. The hypothecated tax and cost allocation models, which add a patient fee to generic medicines prices, are not likely to be socially acceptable.

**Conclusion:** When considering a new pricing model for generic medicines, the impact on innovative medicines and the characteristics of the healthcare system in a given country need to be taken into account. Also, there is a need to continuously follow up the level of competition in off-patent medicines markets and to identify sustainability risks.

## 1 Introduction

Generic medicines play an important role in European healthcare systems and in supporting public health. This is because they generate budgetary savings. For instance, prices of medicines following patent expiry dropped 61% in 14 European countries due to market access of generic medicines (IMS Institute for Healthcare Informatics, 2015). In addition to this, generic medicines improve cost-effectiveness and access to treatment, contribute to medication adherence and patient health (Dylst et al., 2015). By 2018, generic medicines attained a market share by volume of 67% in European retail markets for prescription medicines (IQVIAMIDAS, 2018).

On the one hand, generic medicines companies are under pressure by European pricing policies and *ad hoc* price cuts (Dylst et al., 2013). On the other hand, European generic medicine markets face significant cost increases in the wake of the COVID-19 pandemic and as a result of high inflation and the war in Ukraine (Collis, 2022). Consequently, concerns about the economic sustainability of generic medicines have surfaced and the threat of supply disturbances and shortages of generic medicines is looming. According to a study by the European Commission, shortage notifications have increased and mainly relate to off-patent medicines (Directorate-General for Health and Food Safety European Commission Ecorys et al., 2021). Another study by IQVIA observed that 52%–79% of shortages involve generic medicines (Troein et al., 2020). This tends to be attributed to low-profit margins associated with these products and inadequate pricing policies, although it needs to be recognised that causes of medicines shortages are multifactorial (Affordable Medicines Europe, 2019).

Most European countries regulate generic medicines prices using policy instruments such as external and internal reference pricing, tendering, discounts, clawbacks/paybacks (Dylst and Simoens, 2010; Carone et al., 2012; Vogler and Habimana, 2014). The ability of external reference pricing to restrain costs is limited due to difficulties involved in comparing (net) prices between countries and due to possible strategic behavior of pharmaceutical companies (e.g., launching first in markets with high medicines prices, competing in terms of discounts rather than price) (Carone et al., 2012; Vogler and Habimana, 2014). Internal reference pricing systems have been shown to reduce prices in the short run (Puig-Junoy, 2010), but there is no impact on the increase in pharmaceutical expenditure in the long run (Dylst et al., 2011a). Moreover, these systems may not incite price competition below the reference price (Puig-Junoy, 2010). Tendering for retail prescription medicines has produced short-term savings in the few European countries that have implemented it, but its long-term impact is uncertain (Dylst et al., 2011b). Although discounts, clawbacks/paybacks and extraordinary contributions are effective instruments to generate savings, they negatively affect the economic viability and sustainability of generic medicines supply.

Therefore, the aim of this manuscript is to examine new pricing models for retail generic medicines in Europe with a view to create sustainable competition in the long run. This will ensure that pharmaceutical companies continue to develop and supply generic medicines, and that healthcare payers, physicians and patients benefit from the availability of affordable treatment alternatives.

## 2 Materials and methods

For the purpose of this manuscript, a pricing model refers to all pricing regulations that affect generic medicines companies, starting from setting the price of their products through reimbursement level and patient co-payment up to potential rebates and paybacks.

This qualitative analysis of pricing models for generic medicines was based on a targeted literature review. The review focused on conceptual pricing models and pricing models implemented in practice inside or outside the pharmaceutical sector around the world. With respect to the peer-reviewed literature, the following bibliographical databases were searched: Medline via Ovid, Embase via Ovid, The Cochrane Library, The University of York Centre for Reviews and Dissemination, and EconBiz. The search also encompassed sector-specific peer-reviewed journals that are not indexed such as the Journal of Generic Medicines and the Generics and Biosimilars Initiative Journal. Search terms included: “reference price” and “pharmaceutical”; “reference pricing” and “pharmaceutical”; “generic”, “pharmaceutical” and “reimbursement”; “generic”, “pharmaceutical” and “price competition”; “generic”, “pharmaceutical” and “discount”; and “generic”, “pharmaceutical” and “rebate”. Studies were included if they explored (the impact of) pricing models in the generic medicines market. Given that a previous study of the impact of pricing regulation for generic medicines reviewed the literature until 2009 (Puig-Junoy, 2010), articles published prior to that year were excluded. Furthermore, articles in a language other than English or Spanish were not considered.

The search of the grey literature included websites of international organisations (e.g., OECD, Affordable Medicines Europe, Medicines for Europe, International Generic and Biosimilar Medicines Association), websites of national organisations and industry associations (e.g., Medaxes in Belgium, ABDA in Germany, BOGIN in the Netherlands, APOGEN in Portugal), country reports published by the European Observatory on Health Systems and Policies, pharmaceutical news websites (e.g., The Pharma Letter, The Pink Sheet), and websites of consultancy agencies (e.g., KPMG, Deloitte).

In addition to and based on the literature review, a qualitative study was conducted discussing new pricing models during one-to-one semi-structured interviews and a joint advisory board meeting with five key experts from Belgium, Portugal, Spain, Romania and Greece during the first half of 2022. We acknowledge that the number of experts was small and therefore our results need to be validated in future research. Experts were selected based on their long-standing knowledge of generic medicines markets using purposive sampling and represented countries that vary in terms of generic medicines pricing regulations and market shares, and levels of economic development. Prior to the interviews and the advisory board meeting, experts provided written consent for their participation and oral consent for audio recording of conversations. Interview and meeting records were transcribed and analysed using the QUAGOL guide (Dierckx de Casterle et al., 2012). Access to study records and transcripts is restricted to the research team who need to sign into a storage system by using a 10-character password which is changed every 45 days. Files are stored for 5 years following study completion, after which they are destroyed.

## 3 Results and discussion

The literature search yielded 1,037 records, of which 971 were excluded following screening. Of the 66 full-text articles that were assessed for eligibility, seven articles were included (Value-based pricing, 2011; Puig-Junoy, 2012; Hollis and Grootendorst, 2017; Granlund and Bergman, 2018; Tesar et al., 2021; Medaxes, 2022; Moradpour et al., 2023). In addition, one relevant report from a consultancy agency was identified in the grey literature (Invent, 2020). The following sections and Table 1 present potential pricing models for generic medicines in Europe, discuss their advantages and limitations, and provide examples from around the world. For each of the ten pricing models, the results of the literature review are followed by the main messages and quotes from the expert discussions.

**TABLE 1:** Assessment of potential new pricing models for generic medicines in Europe.

Pricing model	Description	Advantages	Limitations	Examples
Tiered pricing	Model with multiple tiers that set maximum price of generic medicines as a function of number of competitors	More competition results in lower prices	How to set price difference between tiers?	Pan-Canadian Pharmaceutical Alliance Tiered Pricing Framework Pan-Canadian Pharmaceutical Alliance, (2020)
		Stimulates generic market entry through higher price when there is little competition	Need for infrastructure to follow up number of competitors	-Tier 1 if 1 generic priced at 75% or 85% of originator price
				-Tier 2 if 2 generics priced at 50% of originator price
				-Tier 3 if 3 or more generics priced at 25% or 35% of originator price
Price de-linkage from originator medicine	Model which severs the link between the price of the originator medicine and the price of generic medicines	Originator medicine cannot reduce competition by lowering its price to level that is not sustainable for generic medicines	This model does not guarantee competition between originator and generic medicines or among generic medicines	
		Stimulates generic competitors to enter the market		
Automatic indexation	Generic medicines prices are automatically indexed in line with inflation	Higher costs of producing generic medicines are fully passed on in their pricing	This model shifts the whole risk of inflation from companies to the healthcare payer	In Canada, the Patented Medicines Prices Review Board publishes annually the Consumer Price Index Adjustment Factors for patented medicines Patented Medicines Prices Review Board, (2022)
			Increasing generic medicines prices inhibits patient access to treatment	In Germany, the price moratorium in References price-free market segments can be adjusted for inflation Nichtamtliches Inhaltsverzeichnis, (1988)
One-in-one/multiple-out	The cost impact of new regulation is offset by abolishing old regulation(s)	Reduces administrative burden and thus encourages market entry and competition	Difficult to abolish old regulation(s)	This model has been introduced in several European countries and has been proposed by the European Commission Invent, (2020)
			Difficult to calculate cost impact of regulation	
			This is not a pricing model *per se*	
Tax credits	Tax credits are offered to strategically important industries such as generic medicines companies	Supports sustainability of generic medicines markets and guarantees supply of medicines at risk of shortages	How to identify companies and medicines that qualify for tax credits?	Tax credits are given to R&D sectors in the United States, the United Kingdom and France (Invent, 2020; Ministère de l'Econom i.e., des Finances et de la Souveraineté Industrielle et Numérique, 2022)
			Which amount of tax credits generates the required volume of medicines?	
			Companies are not treated equally	
Cost allocation	Additional costs incurred by generic medicines companies are allocated to and paid by patients	Generic companies are fully insulated from cost increases	Increasing generic medicines prices inhibits patient access to treatment and may exacerbate social inequalities	Consumers pay environmental disposal fee charged on some goods Invent, (2020)
Guaranteed margin/fee	Companies earn a guaranteed profit margin on generic medicines	Ensures sustainability of generic medicines markets	This model does not necessarily promote competition	Community pharmacists receive a fixed dispensing fee in Belgium, Denmark and Germany (Invent, 2020; International Pharmaceuti cal Federation FIP, 2021)
			This model requires that drug costs are measured and made transparent	
Hypothecated tax	Specific tax imposed by government is used to support generic medicines companies	Ensures sustainability of generic medicines markets	Same limitations as the cost allocation model	National Insurance Contributions in the United Kingdom GOV.UK, (2023)
			There is a cost involved in collecting and redistributing tax revenues	Life Saving Drugs Program in Australia (Australian Government Department of Health and Aged Care, 2023)
			It is not likely that politicians will levy an extra tax	
Value-based pricing	Set the price at the level that makes a generic medicine cost-effective	Leads to higher prices as it is claimed that generic medicines are currently under-valued	Need to perform a health technology assessment	In England, an innovative medicine is recommended for use if it is cost-effective at the offered price Coombes, (2010)
			Implies that (generic) medicines pricing in a country is based on health technology assessment	
Volume for savings	Generic medicines prices are reduced in return for volume increase	Higher access to generic medicines at lower prices	This model does not in itself promote competition	Price-volume managed entry agreement for innovative medicine
			Higher volume, but lower prices produce mixed impact on sustainability of generic medicines markets	

### 3.1 Tiered pricing

In a tiered pricing model, multiple tiers are established that set a maximum price for generic medicines depending on the number of competitors: if there is little competition, generic medicines market entry is stimulated by allowing a higher price; if there are more competitors, the maximum price is reduced to generate savings. Such a tiered pricing model has, for example, been implemented for generic medicines in Canada since April 2014 (Pan-Canadian Pharmaceutical Alliance, 2020). According to our experts, this model mimics price competition, while ensuring sustainability. However, it is challenging to determine the number of tiers and their maximum price. When the price difference between tiers is small, multiple generic competitors need to enter the market to attain significant price reductions. When there is a large price difference between tiers, there is more margin for companies, but the market entry of relatively few competitors produces substantial price decreases (Hollis and Grootendorst, 2017). Indeed, a recent simulation study showed that the number and spacing of tiers depends on market size and has consequences for welfare and prices (Moradpour et al., 2023). Moreover, to operationalise a tiered pricing model, infrastructure needs to be developed to follow up the number of competitors, their market entry and exit.

“*I think tiered pricing is a more complex system, it increases the administrative burden, and the national healthcare insurance has to follow up each of these different molecules or markets within their country and have to collect data on the level of suppliers, prices and so on. You also have to set up an IT infrastructure. It's a very appealing model and in practice, I think all of these barriers can and should be overcome.*”

Finally, a tiered pricing model can be implemented in the context of existing generic medicine price regulation. For example, under the internal reference pricing system in Germany, reference prices were adjusted based on the level of competition (although this is no longer the case now) (Danzon and Ketcham, 2004).

### 3.2 Price de-linkage from originator medicine

Several European countries (such as Austria, Bulgaria, Finland, France, Ireland, Italy, Poland and Spain in 2020) set the price of generic medicines as a percentage below the originator price (Medicines for Europe, 2021). This pricing model provides an incentive for the originator medicine to reduce its price to a level that is unsustainable for generic medicines and, as a consequence, force generic competitors to leave the market (Simoens and De Coster, 2006). This can be avoided by de-linking the price of generic medicines from that of the originator medicine. Multiple variants of the price de-linkage model can be applied. Under full de-linkage, the link between the originator price and the price of generic medicines is completely cut. Under partial de-linkage, the originator price is used as reference when establishing prices of generic medicines entering the market. Any subsequent changes in the price of the originator medicine do not impact prices of generic competitors. Under competition maturity de-linkage, generic medicines prices cease to be linked to the originator price once competition reaches a certain level of maturity related to the number of competitors and their market shares.

Our experts shared the opinion that price linkage restrains the development of sustainable generic medicines markets in Europe and that price de-linkage from the originator medicine is a feasible model to remedy that situation. However, they also argued that its application might be limited to markets characterised by high volume and the presence of multiple generic competitors. Competition is unlikely to be stimulated by price de-linkage from the originator medicine in markets with few generic medicines suppliers.

“*I think that it is probably the easiest measure to implement among the set of measures that you are proposing. De-linkage makes sense according to the level of competition that you have in a certain market.”*

*“I think that such a model could only be applied to countries or to molecules with a large volume. If you only have one or two suppliers in the market, we know that competition will not happen.”*

### 3.3 Automatic indexation

In this model, generic medicines prices are automatically adjusted for inflation. This ensures that companies are insulated from the increasing costs of goods and services in the economy, and thus avoids the negative impact of inflation on the sustainability of generic medicines markets. An example of this model from outside the pharmaceutical sector is the automatic indexation of wages and pensions in Luxembourg (myLIFE, 2018) and Belgium. This model can be introduced on its own or in combination with other pricing models.

An automatic indexation model implies that the risk of inflation is transferred in full from companies to the healthcare payer. To increase the acceptability of this model to healthcare payers, they could negotiate a risk-sharing arrangement with companies, agreeing that generic medicines prices are adjusted for a fraction of the inflation rate. This fraction, in turn, depends on how much risk associated with inflation a healthcare payer is willing to accept in order to prevent market withdrawals.

### 3.4 One-in-one/multiple-out

The implementation of new regulation may increase operational costs of generic medicines (an example of this is the EU Falsified Medicines Directive (Medicines for Europe, 2020)). In this context, the one-in-one/multiple-out model implies that the introduction of a new regulation is accompanied by the removal of one or multiple existing regulations, so that the net cost impact is zero or even negative (Van den Abeele, 2021). This model has already been applied in a number of European countries (e.g., Italy, Latvia, Lithuania and Spain) and has been proposed for use by the European Commission (Invent, 2020).

The one-in-one/multiple-out model encourages decision and policymakers to consider the cost impact of new and old regulations, although it is not straightforward to determine this impact or to identify regulations that can be abolished. Our experts did not perceive the one-in-one/multiple-out model as a pricing approach *per se* but rather as an additional tool that can be used to “cut the red tape” and hence incite generic companies to enter the market. Simplification of procedures might be a first step towards enhancing the sustainability and competitiveness of generic medicines’ markets.

*“I agree with the principle one-in-one-out to reduce the administrative burden. I fully support that, but I am a bit sceptical about whether this will happen in practice. If you look at regulation and the administrative burden related to generic medicines, that only increases over time.”*

### 3.5 Tax credits

Tax credits can be used by governments as an instrument to support strategically important industries. For instance, the United States, the United Kingdom and France have a tradition of providing tax credits to the R&D sector (Invent, 2020; Ministère de l'Econom i.e., des Finances et de la Souveraineté Industrielle et Numérique, 2022). This model could be envisaged for the generic medicines industry in general or for generic companies that supply medicines at risk of shortages due to low economic sustainability in particular.

This model provides some level of flexibility and adjustment towards dynamic market changes. However, a number of challenges arise with its implementation (Invent, 2020). Agreement needs to be sought on which medicines are at risk of shortages and which generic companies are granted tax credits. It is also difficult to set the amount of tax credit at a level that produces the required volume of medicines (and avoids under- or over-supply). Furthermore, tax credits may have differing significance to companies with different portfolios.

Our experts pointed out that pricing models like tax credits which interfere with national taxation systems may raise questions about their legality or compliance with already existing tax arrangements. Moreover, conflict of interest, lack of transparency, accusations of unequal treatment of companies by the government, low political support and implementation difficulties were seen as obstacles for introducing tax credits to generic medicines markets.

*“I think there are opportunities within the European legislation to have some level of discrimination for a temporary time in order to avoid, for example, shortages as a public health crisis, but then you need to reflect on the legal provisions and the European legislation on how and to what extent you can accommodate for this.”*

*“Tax credits might be difficult and might not achieve the goal because it's taken just as an opportunity..no reason to believe that these tax credits will be used for investment.”*

### 3.6 Cost allocation

In general, this model allocates costs of a public good (arguably such as medicines) to the different stakeholders in the supply chain (i.e., pharmaceutical companies, wholesalers and pharmacies) in a proportional manner according to their contributions and responsibilities in the process (Invent, 2020). In practice, a fixed end-user (patient) fee is added to medicine prices to cover increased operational costs of generic medicines suppliers. A third party is established to transfer gathered funds to the suppliers concerned. An example of end-user fees is the environmental charge levied on goods in some industries.

This pricing model which imposes an additional patient co-payment has been appraised by our experts as having a very low implementation potential in Europe, except for countries such as Denmark, Sweden or Norway that have a long-standing tradition of collective responsibility and public goods. Also, the additional financial burden of acquiring medicines may deepen social inequalities.

*“Cost allocation is probably applicable in Denmark or Sweden or Norway. In Central and Eastern Europe, we hate the concept of collective property.”*

### 3.7 Guaranteed margin/fee

This pricing model ensures that generic companies receive a certain profit margin by applying a fee above the production and distribution costs of medicines (Invent, 2020). This provides a flexible solution given that the fee can be adjusted to changing market dynamics (such as the number of competitors). The amount of the margin/fee depends on, amongst other things, product characteristics. However, the operationalisation of this pricing model requires transparency about production and distribution costs of medicines, and consensus on how to measure costs. The guaranteed margin/fee model can be exemplified by the payment of a fixed dispensing fee to community pharmacists in some countries (International Pharmaceuti cal Federation FIP, 2021).

Although our experts agreed that this pricing model improves the sustainability of generic medicines, they also argued that it does not contain incentives to bolster competition or for companies to reduce costs.

*“I think this pricing model addresses the concerns of the generic industry in terms of economic viability or guarantees that. However, I think we need to supplement it with incentives for competition because I do not see any incentives within this model. And I share the concerns about operationalizing. Manufacturers would need to disclose some cost data and that is always a very sensitive issue.”*

### 3.8 Hypothecated tax

In general, the hypothecated tax model entails that the government collects tax revenues for a specific purpose. In our case, these tax revenues would be allocated to generic companies as a form of supportive financial subsidy. Examples of this model include the National Insurance Contributions in the United Kingdom which serve to pay for social security benefits (GOV.UK, 2023), and the Life Saving Drugs Program in Australia which allows to reimburse orphan drugs that are deemed to be not cost-effective (Australian Government Department of Health and Aged Care, 2023).

This pricing model suffers from the same limitations as the cost allocation model (cfr. supra). Also, in practice, it is not clear what the level of the hypothecated tax should be to maintain sustainable competition in European off-patent markets. Moreover, the process of collecting and allocating tax revenues to companies involves a cost and it may be challenging to set criteria to redistribute these funds.

*“You will not be able to convince politicians and decision makers that they have to announce on television that from tomorrow onwards, all patients will need to pay an additional co-payment because we consider healthcare to be a public good. And they would argue, we already pay through our taxes for healthcare and there is just no rationale for increasing prices.”*

### 3.9 Value-based pricing

Value-based pricing, a model typically proposed for innovative medicines, sets the price of a medicine so that the benefits provided by the medicine justify its costs (Value-based pricing, 2011). Implementing value-based pricing for generic medicines is a new concept inspired by the claim of our experts that, as a result of continuous price erosion, generic medicines prices have dropped below the level that makes them cost-effective and hence these medicines are under-valued.

*“Healthcare payers and health technology assessment agencies should re-assess the value of a medicine once it becomes off-patent, and some of these medicines have been on the market for a very long time. The price has decreased, but they are actually very valuable medicines, maybe even more valuable than some more expensive innovative therapies. I suspect that if we look at the cost-effectiveness of a generic medicine, which has a much lower price and maybe generates similar effectiveness, and compare it with the cost-effectiveness of these innovative medicines, then we are actually as a society better off with using these valuable old generic medicines.”*

Our experts regarded the application of this pricing model to generic medicines more as a strategic mindset changer by switching the narrative from “generics provide savings” to “generics provide treatment benefits at exceptionally affordable cost”. They also noted that a major implementation challenge for the value-based pricing model is the need to perform value assessments in countries with limited health technology assessment capacity and/or expertise (especially if this would be applied to a whole therapeutic class including all available treatment options). Finally, it is clear that this model is only feasible in countries that draw on health technology assessment to price (and reimburse) medicines.

### 3.10 Volume for savings

The likely existence of economies of scale in the generic medicines industry makes it possible to produce a greater quantity of generics at a lower cost. Therefore, this pricing model proposes that, if the volume share of generic medicines increases in year X as compared to year X-1, companies give back a certain percentage of the resulting increased turnover in year X+1 (Medaxes, 2022). This model incites governments to introduce demand-side measures promoting use of generic medicines, while guaranteeing savings. Generic medicines companies benefit from increased uptake, but accept that part of this growth is “clawed back”. A drawback of this pricing model according to our experts is that it does not necessarily boost competition. Additionally, its implementation requires the government and industry to agree on the target volume increase and associated price decrease of generic medicines. Finally, achieving the target volume increase in practice might prove challenging and may depend on the specificity of the pharmaceutical market.

*“We need to take into account the generic market share in every country because there is something different if it’s 30% or if it's 70% when we talk about volume. The volume situation is also different in therapeutic areas or groups.”*

## 4 Conclusion

This study has reviewed a variety of new pricing models for generic medicines in Europe. It is clear there is no single optimal model which can be universally implemented. Instead, we advocate that, when discussing the implementation of a new generic medicines pricing model in a country, decision and policymakers take a holistic approach. Such an approach considers both general issues (e.g., healthcare system features, impact of existing medicines pricing policies, attitudes towards generic medicines, consequences for innovative products) and market-specific issues (e.g., market size, number of suppliers and their market shares by volume). Also, the implementation of a new pricing model for generic medicines may incite stakeholders to call for revisiting pricing models for originator medicines (or other health technologies).

Based on the literature review and expert opinion, this study identified tiered pricing, price de-linkage from originator medicine and automatic indexation as having the best implementation potential for generic medicines. Models involving an additional patient fee (i.e., hypothecated tax and cost allocation) were uniformly considered as having very low social acceptability. The other pricing models (i.e., one-in-one/multiple-out, tax credits, value-based pricing, volume for savings and guaranteed margin/fee) were assessed as being less feasible.

Finally, a crucial step towards sustaining competition in European off-patent medicines markets is to start consistently monitoring the level of competition (by means of indicators such as the Herfindahl-Hirschman Index) and to take action when deterioration of competition is considered a threat to continued supply.

## Data Availability

The original contributions presented in the study are included in the article/Supplementary Material, further inquiries can be directed to the corresponding author.
